# Benefit framing, personality traits and intention to adopt a flexitarian diet

**DOI:** 10.3389/fnut.2026.1848757

**Published:** 2026-10-15

**Authors:** Lory Barile, Florian Kollmar, Andreas Markoulakis

**Affiliations:** Department of Economics, University of Warwick, Coventry, United Kingdom

**Keywords:** behaviour change, Big Five, flexitarian diet, information nudge, sustainable food

## Abstract

**Introduction:**

Shifting diets towards more plant-based patterns is important for public health and environmental sustainability, yet evidence on the effectiveness of behavioural interventions remains mixed. This study examines whether benefit framing influences willingness to adopt a flexitarian diet and whether responses vary by personality and socio-demographic characteristics.

**Methods:**

In a between-subjects online experiment, 412 UK adults were randomly assigned to financial, health, environmental, or control conditions; 303 participants were included in the final analytical sample. Probit models with interaction terms and average marginal effects were estimated.

**Results:**

Health framing increased the probability of expressing a positive adoption intention by approximately 17 percentage points relative to the control condition, whereas financial and environmental framing had no statistically significant overall effects. Environmental morale and willingness to take risks were also positively associated with adoption intention. In the control condition, a one-standard-deviation increase in openness, and conscientiousness was associated with 12.8 and 9.1 percentage-point higher probabilities of a positive intention, respectively. Conditional treatment-effect analyses suggest a ceiling effect: among participants one standard deviation below the mean, health framing increased intentions by 36.5 percentage points for openness and 32.9 percentage points for conscientiousness, while environmental framing increased intentions by 25.5 percentage points for openness; these effects were not statistically significant at higher trait levels.

**Discussion:**

These findings indicate that the effectiveness of benefit-based messages may depend not only on message content but also on individual characteristics and suggest that more differentiated communication strategies may improve the relevance of sustainable-diet interventions.

## 1 Introduction

Dietary choices play a central role in shaping both human health and environmental sustainability. Diets high in red and processed meat and low in fruits and vegetables are associated with increased risks of chronic diseases, including cardiovascular disease, diabetes, and certain cancers, and are expected to become more prevalent in the absence of intervention (1, 2). In the United Kingdom alone, unhealthy diets are estimated to contribute to more than 75,000 premature deaths annually, highlighting the substantial public-health burden associated with current dietary patterns (3). At the same time, food production contributes significantly to environmental degradation, accounting for approximately 15–30% of global greenhouse gas emissions, occupying around 40% of land use, and consuming the majority of freshwater resources (2, 4). These combined pressures have placed dietary change at the centre of both public health and climate policy debates.

Within this context, flexitarian diets have emerged as a potentially realistic pathway for reducing the health and environmental impacts associated with current consumption patterns. A flexitarian diet emphasises predominantly plant-based foods while allowing moderate consumption of animal products, thereby offering a more flexible alternative to fully vegetarian or vegan diets. Evidence suggests that such diets can substantially reduce greenhouse gas emissions, resource use, and diet-related mortality, while also being economically accessible in high-income contexts (2, 5). By retaining elements of existing consumption patterns, flexitarian diets may also be more acceptable and scalable among the general population (6). Despite these potential benefits, however, widespread adoption of more plant-based dietary patterns remains limited (7).

This limited uptake raises an important behavioural question: how can individuals be encouraged to consider adopting a flexitarian diet? Information-based interventions represent one relatively low-cost approach to promoting dietary change and commonly communicate the health, environmental, ethical, or other benefits associated with particular dietary choices. However, individuals may also perceive risks or uncertainties associated with dietary transition. In the case of plant-based foods and alternatives, these may include concerns about food safety, such as exposure to mycotoxins, pesticide residues, and processing contaminants (8). Such perceived risks may therefore act as an additional barrier to adoption, despite receiving considerably less attention than the potential benefits of plant-based and flexitarian diets (9). In the present study, because the intervention provides information but does not directly alter participants’ food environment or observe subsequent dietary behaviour, behavioural intention provides the most immediate outcome through which responses to the intervention can be evaluated.

A large body of research has examined whether information and other behavioural interventions, such as communicating health, environmental, or ethical benefits, can influence dietary choices. Many studies of sustainable food consumption draw on the Theory of Planned Behaviour (TPB), which conceptualises behavioural intention as a proximal antecedent of behaviour and emphasises the role of attitudes, subjective norms, and perceived behavioural control in shaping intention (4, 10–12). This theoretical framework further supports the use of behavioural intention as the primary outcome in the present study. Nevertheless, the well-documented intention-behaviour gap means that changes in stated intention should not be interpreted as equivalent to subsequent behavioural change (13, 14).

Experimental evidence suggests that benefit-based messaging can influence attitudes and intentions, although effects are often modest and highly context-dependent (13, 15, 16). More broadly, interventions aimed at promoting healthier and more sustainable diets range from structural measures, such as taxes, subsidies and regulation, to behavioural approaches involving framing, social norms, nudging, feedback, labelling and changes to the choice environment (17–20). Although structural interventions may generate stronger behavioural responses, they can also be politically contentious, particularly when directed at reducing meat consumption (21, 22). Consequently, comparatively light-touch approaches based on information provision and behavioural insights^1^ have become increasingly prominent. However, their effectiveness remains uncertain. In particular, studies comparing health and environmental messages frequently report small or statistically insignificant effects, and combining several benefit messages does not necessarily increase their effectiveness (16).

One comparatively underexplored dimension of this literature is the role of financial benefits. Financial considerations have been shown to influence pro-environmental behaviour in other domains, including energy efficiency and green technology adoption (23, 24), yet considerably less is known about whether cost-related information affects dietary intentions. This represents an important gap given evidence that flexitarian diets may be economically accessible, and potentially more affordable than conventional diets, in high-income countries (5), despite common perceptions to the contrary (25). Comparing financial framing directly with more commonly studied health and environmental framings therefore provides an opportunity to examine whether different types of perceived benefit generate different behavioural responses.

A further limitation of focusing only on average treatment effects is that behavioural responses to information are unlikely to be uniform. Individuals differ in their values, motivations, beliefs, preferences, and personal characteristics, which may influence both their underlying willingness to adopt more sustainable diets and their responsiveness to particular messages. Previous research has documented heterogeneity in sustainable food choices according to values, motivations, and prior beliefs (4, 26, 27, 68), highlighting the limitations of “one-size-fits-all” approaches and the potential value of audience segmentation (12). However, the role of relatively stable psychological characteristics in shaping responses to benefit framing remains less well understood.

The Big Five personality framework provides a well-established and widely validated approach to capturing such individual differences. Rather than selecting isolated psychological characteristics on an *ad hoc* basis, the framework captures five broad and relatively stable personality domains (i.e., openness, conscientiousness, extraversion, agreeableness, and neuroticism) that have been linked to dietary and pro-environmental behaviour. Personality may influence food choices through characteristics such as self-discipline, responsiveness to external cues, openness to new experiences, concern for health, and emotional tendencies. In particular, openness has emerged as an important predictor of vegetarian and vegan dietary patterns (28, 29), while agreeableness and conscientiousness have also been associated with healthier and more sustainable dietary choices (30–32). Neuroticism and extraversion have likewise been linked to eating behaviour, although these relationships appear more complex and may operate through emotional eating, sensitivity to external food cues, and social influences (33, 34).

In the present study, the TPB provides the overarching framework for conceptualising behavioural intention as the proximal outcome of interest, while personality traits and other individual characteristics are incorporated as complementary sources of heterogeneity. We therefore adopt a TPB-informed extended framework rather than attempting to estimate the complete structural TPB model. Within this framework, personality traits are treated as relatively stable characteristics that may be associated with baseline behavioural intentions and may also moderate individuals’ responsiveness to different benefit framings. This perspective is complementary to broader social-cognition approaches, including frameworks such as COM-B, which have also been applied to food-related behaviour (35, 36). The purpose of incorporating personality is therefore not to replace these broader frameworks, but to examine whether stable individual differences help explain variation in responses to behavioural interventions.

Against this background, this study examines how three distinct benefit framings (i.e., financial, health, and environmental) affect behavioural intentions to adopt a flexitarian diet and whether these effects vary across individuals. The study addresses three related gaps in the existing literature. First, although health and environmental framings have received considerable attention (37, 38), financial framing remains comparatively underexplored in the context of dietary change. Second, relatively few studies directly compare financial, health, and environmental benefit messages within the same experimental design; where three framings have been compared, they have often involved different dimensions, such as financial, health, and social-norm messages (39). Third, while behavioural responses to sustainable-food interventions are increasingly recognised as heterogeneous, the extent to which personality traits interact with responses to different benefit framings remains insufficiently understood (40, 41).

We address these gaps using a between-subjects online experiment in which UK adults were randomly assigned to a financial, health, environmental, or control condition. We examine both the average effects of benefit framing and heterogeneity in these effects according to personality traits and selected attitudinal variables and socio-demographic characteristics. In doing so, the study contributes to a growing literature emphasising the importance of moving beyond average treatment effects when designing and evaluating behavioural interventions (42).

The results indicate that health framing increases the probability of expressing a positive intention to adopt a flexitarian diet, whereas financial and environmental framing do not have statistically significant overall effects. We also find evidence that behavioural intentions vary according to environmental attitudes, risk preferences, and selected personality traits, and that some personality-related associations differ across framing conditions. These findings highlight the importance of behavioural heterogeneity and suggest that more differentiated, behaviourally informed communication may therefore help improve the relevance of policy messages across consumers and other actors in the food system, although the effectiveness of such approaches requires further testing (43).

The paper is organised as follows. Section 2 presents details of the experimental design, research questions, and methods used to conduct the analysis. Section 3 reports regression results. Section 4 provides some discussion of results and concluding remarks.

## 2 Methods

### 2.1 Experimental design

The study employs a between-subjects experimental design in which participants were randomly assigned to one of four experimental conditions, implemented through vignette-based scenarios.^2^ Participants were provided with a standardised definition of a flexitarian diet based on Springmann et al. (5), ensuring consistency across treatment conditions. Participants in the treatment groups additionally received condition-specific information corresponding to financial, health, or environmental benefits. These conditions varied the type of information provided about the consequences of switching from a benchmark diet to an energy-balanced flexitarian diet, drawing on evidence from Springmann et al. (2, 5). The four conditions were: (1) control, in which no additional information was provided; (2) financial, which highlighted potential annual cost savings; (3) health, which emphasised reductions in premature mortality risk; and (4) environmental, which presented reductions in diet-related CO₂ emissions. All vignettes used in the experiment are provided in Supplementary Appendix A.

The primary outcome for our analysis is the behavioural intention to adopt a flexitarian diet. This was inferred using a question adapted from Laroche et al. (44): *“I would agree to take on a flexitarian diet starting today.”* Responses were recorded on a 5-point Likert scale ranging from “strongly disagree” to “strongly agree.” “Agree” and “strongly agree” responses were then aggregated to form a discrete variable for the empirical analysis (taking values 1 = likely to adopt a flexitarian diet; and 0 otherwise).

### 2.2 Hypotheses tested

Using this experimental design, our study addresses two main research questions: (i) which benefit framing is most effective in increasing behavioural intention to adopt a flexitarian diet; and (ii) how personality traits and socio-demographic characteristics are associated with behavioural intention and responsiveness to benefit framing.

In answering these questions, the paper investigates two complementary dimensions of heterogeneity. First, it examines how the Big Five personality traits relate to behavioural intentions to adopt a flexitarian diet. Consistent with prior evidence, openness, agreeableness, and conscientiousness are expected to be positively associated with pro-environmental attitudes and behaviours (32). Neuroticism and extraversion may also be relevant to dietary behaviour through their associations with eating styles and, indirectly, food choices (33). Second, and as a more novel contribution, the study explores whether personality traits moderate responsiveness to different benefit framings. Insights from behavioural and environmental psychology suggest that individuals lower in agreeableness (often associated with more egoistic value orientations) may respond more strongly to self-benefiting (financial) messages (27, 45), whereas individuals higher in agreeableness may be more responsive to environmental framing (46). Similarly, conscientiousness has been consistently associated with healthier dietary and health-related behaviours (30, 47), suggesting that health-related benefits may be particularly salient to individuals higher in this trait.

In addition to personality traits, the analysis considers whether socio-demographic characteristics shape responsiveness to benefit framing. This responds to calls in the literature to identify interventions that are effective across different population groups, particularly among less affluent households (41). In particular, financial framing may be especially salient for lower-income individuals if it reduces perceived cost barriers to dietary change, while health framing may resonate more strongly with older individuals as health-related outcomes become increasingly salient (48).

In this way, rather than focusing solely on average treatment effects due to benefit framing, the analysis provides a more nuanced assessment of how behavioural interventions operate across heterogeneous groups. As indicated in the introduction, this is particularly relevant given the mixed evidence on the effectiveness of health and environmental messaging (1, 16), alongside the relative neglect of financial framing in dietary contexts despite its potential relevance (5, 25).

This translates into seven hypotheses:

*H1:* Benefit framings (financial, health, environmental) increase behavioural intentions relative to control.

*H2:* Big Five personality traits are associated with behavioural intentions to adopt a flexitarian diet.

*H3:* Financial framing is more effective among individuals lower in agreeableness.

*H4:* Environmental framing is more effective among individuals higher in agreeableness.

*H5:* Health framing is more effective among individuals higher in conscientiousness.

*H6:* Financial framing is more effective among lower-income individuals.

*H7:* Health framing is more effective among older individuals.

While this study does not empirically measure the individual constructs of the Theory of Planned Behaviour [TPB; (10)], TPB provides the overarching conceptual framework for understanding how the information intervention may influence behavioural intention. Specifically, the benefit-framed messages are conceptualised as external informational prompts that may alter individuals’ beliefs about the expected consequences of adopting a flexitarian diet and their evaluations of those consequences. Health and environmental messages may primarily influence beliefs about the corresponding benefits of dietary change, while financial information may additionally affect perceptions of affordability and potential barriers to adoption. Within the TPB framework, such changes in beliefs and evaluations may contribute to the formation of behavioural intentions, which in turn represent the proximal antecedent of subsequent behaviour. Because these intermediate TPB constructs and actual dietary adoption are not directly measured in the present study, this pathway is interpreted as the theoretical mechanism underpinning the intervention. Figure 1 summarises this conceptual framework and the relationships examined in the study.

**Figure 1:**
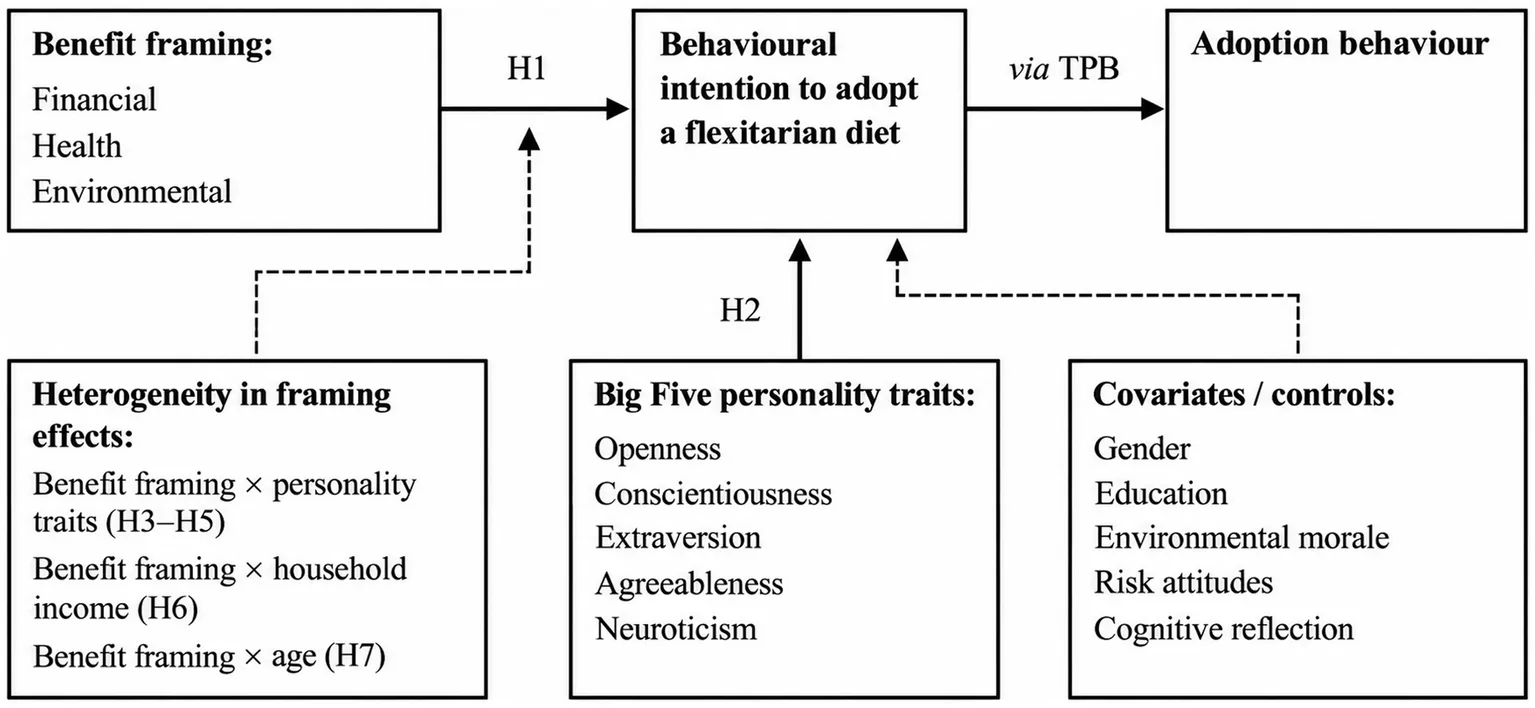
Conceptual research model.

Figure 1 illustrates the main empirical relationships and sources of heterogeneity considered in the analysis. Benefit framing is hypothesised to influence behavioural intention (H1), while Big Five personality traits are examined both in relation to behavioural intention (H2) and as a source of heterogeneity in responses to the different benefit framings (H3–H5). Additional heterogeneity is examined across household-income and age groups (H6–H7), while gender, education, environmental morale, risk attitudes, and cognitive reflection are included as covariates. The pathway from behavioural intention to adoption behaviour reflects the theoretical relationship proposed by TPB because actual dietary adoption is not directly observed or analysed in this study.

### 2.3 Procedure and participants

The experiment was conducted online in June 2025 using Qualtrics, with participants recruited through Prolific. Eligibility for participation was restricted to UK residents aged 18 years or older, consistent with Prolific’s participation requirements. The adult sample was also appropriate given the study’s focus on individuals’ autonomous dietary choices.

The study was advertised under the title “Flexitarian Diet.” After providing informed consent, participants first completed the Big Five personality questionnaire, followed by an instructional manipulation check. All participants were then provided with the same standardised definition of a flexitarian diet, based on Springmann et al. (5), to ensure a common understanding of the dietary pattern. Participants were subsequently randomly assigned, using Qualtrics randomisation tools, to one of four experimental conditions: control, financial, health, or environmental. The control group received no additional benefit information, while the three treatment groups received information highlighting, respectively, the financial, health, or environmental consequences of adopting a flexitarian diet. Following exposure to the assigned condition, participants reported their behavioural intention to adopt a flexitarian diet and then completed the remaining socio-demographic and attitudinal questions, together with a second instructional manipulation check. The ordering of sections and questions ensured that the personality measures were completed before treatment exposure, thereby avoiding potential contamination of these measures by the experimental manipulation.

Participant recruitment was conducted within a fixed project budget, which determined the maximum attainable sample size. A total of 412 participants took part in the survey. To allow consistency and comparability across respondents, responses were excluded if participants failed to complete the survey (*n* = 8) or failed both instructional manipulation checks (*n* = 4), following established protocols (49, 50). Following this initial quality screening, the sample comprised 400 participants.

To ensure alignment with the research objective of examining the willingness to adopt a flexitarian diet, the analysis focused on participants not currently following a specific dietary regime. This information was captured in the experimental follow-up questionnaire. Individuals adhering to structured diets (e.g., vegetarian or vegan) are likely to exhibit limited scope for behavioural change and systematically different preferences, which could confound the estimated effects of benefit framing [see, e.g., (2)]. This restriction yielded a sample of 312 individuals. After accounting for missing responses and “prefer not to say” answers, the final analytical sample comprised 303 participants.^3^ Participants were compensated at Prolific’s standard rate and received an additional £1.50 bonus upon completion of the experiment, which had an average duration of approximately 12 min.

The survey collected a range of individual characteristics, including socio-demographic variables such as age, gender, education, occupation, household income, ethnicity and area of residence, as well as attitudinal measures including risk attitudes, environmental morale and political orientation. Consistent with standard practise in experimental research, participants also completed a cognitive reflection test (51), with recent evidence further supporting cognitive reflection as a distinct and measurable construct (52), and two instructional manipulation checks. The socio-demographic measures were adapted from the 2021 UK Census, while the attitudinal measures were drawn from the European Values Survey for environmental morale and, for risk attitudes, from Dohmen et al. (53), and were supplemented with pre-screening information available through Prolific. These data were collected to support the empirical analysis; the subset of variables included in the reported regression models is described in Section 2.4.

Personality traits were measured using the Big Five Inventory-10 (BFI-10) (54), a validated short-form instrument capturing the five broad personality domains: extraversion, agreeableness, conscientiousness, neuroticism, and openness to experience (55). We selected the BFI-10 to minimise respondent burden within the wider online experiment. As a validated short-form measure, it provides an efficient assessment of the five broad personality domains with acceptable reliability and validity, while longer inventories offer more detailed trait measurement (54). Each trait was assessed using two items on a 5-point Likert scale ranging from “strongly disagree” to “strongly agree” (with 1 = strongly disagree; and 5 = strongly agree). The questionnaire is available in Supplementary Appendix A.

### 2.4 Data

The sample construction processes described above and missing responses (e.g., “prefer not to say”) leave a final analytical sample of 303 observations. Table 1 summarises the definition and measurement of all variables included in the empirical analysis.

**Table 1:** Description of variables.

Variable	Definition	Measurement
Intention to adopt a flexitarian diet	Respondent’s agreement with intention to adopt a flexitarian diet.	Main analysis: 1 = agree/strongly agree; 0 = otherwise.
Benefit framing	Experimental condition.	Control; Financial; Health; Environmental.
Gender	Self-reported gender.	1 = Male; 2 = Female.
Age	Respondent age.	1 = 18–34; 2 = 35–44; 3 = 45–54; 4 = 55+. *Base category*: 18–34.
Education	Respondent highest educational attainment.	From 1 = Up to secondary to 4 = Postgraduate. *Base category*: Up to secondary.
Household income	Annual household income.	From 1 = <£25 K to 4 = £75 K +. *Base category*: <£25 K.
Environmental morale	Index; derived from willingness to pay to protect the environment.	1–5 Likert scale; higher values indicate stronger environmental morale.
Cognitive reflection	Cognitive reflection test scores.	0–3; higher values indicate greater number of correct responses and greater cognitive reflection.
Risk attitudes	General willingness to take risks.	0–10; higher values indicate greater willingness to take risks.
Big Five personality traits	Personality traits created using BFI-10.	Two items per personality trait; 1–5 Likert scale. Trait scores are standardised to mean = 0 and SD = 1.

The dependent variable is a binary indicator of behavioural intention to adopt a flexitarian diet, coded as 1 for respondents who agreed or strongly agreed with the intention statement, and 0 otherwise. To ensure sufficient observations across treatment conditions, several demographic categories are collapsed into broader groups for the empirical analysis, as detailed in Table 1. Specifically, the lower (i.e., 18–24 and 25–34) and upper (i.e., 55–64 and 65+) age groups are combined into “18–34” and “55+”; the first two education categories (i.e., 1 = Primary school and 2 = Secondary up to 16) are consolidated into “Up to Secondary”; and household income brackets are grouped into broader categories at the lower (<£10 K and £10 K-£24,999) and upper (£75 K-£99,999; £100 K-£124,999 and >£125 K) ends to form categories “<£25 K” and “£75 K+” for the regression analyses. Categories with no observations (e.g., non-binary gender) and “prefer not to say” responses are excluded and coded as missing values.^4^ Given limited variation in some controls and the focus of our study on heterogeneity in behavioural responses, the empirical analysis includes a specific set of covariates: gender, age, education, and income as socio-demographic controls, and environmental morale, risk attitudes, and cognitive reflection test scores as attitudinal measures.

Moving to attitudinal variables, risk attitudes are measured on a 0–10 scale following Dohmen et al. (53), using a self-assessment question asking respondents to rate their general willingness to take risks on a scale from 0 to 10. More recent evidence supports the stability and predictive validity of stated general risk preferences across behavioural domains (56). Higher values indicate greater willingness to take risks, while lower values indicate greater risk aversion. Environmental morale is captured using two items from the European Values Survey [see also (46)], which measure respondents’ willingness to contribute financially to environmental protection. Responses are recorded on a 5-point Likert scale (1 = strongly disagree; 5 = strongly agree) and averaged to construct an environmental morale index, such that higher values indicate stronger pro-environmental attitudes. The index ranges between 1 and 5 and exhibits good internal consistency (Cronbach’s alpha 0.82).

Finally, as indicated in Section 2.3, personality scores are measured using the BFI-10, which captures five broad dimensions of personality: extraversion, agreeableness, conscientiousness, neuroticism, and openness to experience. Personality scores are constructed from responses to items measured on a 5-point Likert scale (1 = strongly disagree; 5 = strongly agree), with reversed items inverted prior to aggregation.^5^ The resulting domain scores are then standardised (mean = 0, SD = 1) to place all traits on a common scale. This is done to improve comparability of coefficients and reduce potential scaling issues which would otherwise make the interpretation of interaction terms included in the empirical regression below (see section 3) difficult. Full details of variable construction, coding and measurement scales are reported in Table 1.

Table 2 presents descriptive statistics for the variables used in the empirical analysis (*N* = 303). Overall, 45.9% of participants included in the final sample report a positive intention to adopt a flexitarian diet. Assignment across treatment conditions is well balanced, with each condition comprising roughly one quarter of the sample. The sample is broadly diverse in terms of age and income, although it is skewed towards higher educational attainment, with nearly 50% holding a college or university degree. Attitudinal measures indicate moderate pro-environmental preferences and cognitive abilities. Environmental morale averages 2.97 on a 1-5-point scale, while the mean cognitive reflection score is 1.87 (out of 3), suggesting a moderate-to-high level of reflective thinking. Furthermore, with a sample mean of 4.73, participants in our study are, on average, slightly risk-averse to risk-neutral.

**Table 2:** Descriptive statistics.

Variable	*N*	Mean	SD	Min	Max
Intention to adopt a flexitarian diet	303	0.459	0.499	0	1
Control	71	0.234	0.424	0	1
Financial	76	0.250	0.434	0	1
Health	80	0.264	0.441	0	1
Environmental	76	0.250	0.434	0	1
Gender	303	1.455	0.499	1	2
Age	303	2.393	1.145	1	4
*Age 18–34 (base)*	88	0.290	0.454	0	1
Age 35–44	82	0.270	0.445	0	1
Age 45–54	59	0.194	0.396	0	1
Age 55+	74	0.244	0.430	0	1
Education	303	2.743	0.902	1	4
*Up to secondary (base)*	38	0.125	0.331	0	1
Further education	58	0.191	0.394	0	1
College/University	151	0.498	0.500	0	1
Postgraduate	56	0.184	0.388	0	1
Income	303	2.304	0.987	1	4
*Income <25 K (base)*	71	0.234	0.424	0	1
25 K-49,999	113	0.372	0.484	0	1
50 K-74,999	75	0.247	0.432	0	1
75 K+	44	0.145	0.352	0	1
Environmental morale	303	2.970	1.156	1	5
Cognitive reflection (test score)	303	1.868	1.175	0	3
Risk attitudes	303	4.733	2.367	0	10
Extraversion	303	0	1	−1.655	2.112
Agreeableness	303	0	1	−2.753	1.552
Conscientiousness	303	0	1	−3.503	1.249
Openness	303	0	1	−2.606	1.531
Neuroticism	303	0	1	−1.580	1.983

Heterogeneity is examined using three separate probit specifications. The first specification interacts the three treatment indicators with the five standardised personality traits. The second specification interacts the treatment indicators with household-income categories, while the third interacts the treatment indicators with age categories. Each model includes the relevant lower-order terms and the set of control variables described above. Estimating these specifications separately reduces the number of parameters estimated in any one model and allows each dimension of heterogeneity to be examined independently. The treatment-by-personality specification is reported in Table 3 and Supplementary Table A1. For the income and age specifications, results are reported in Supplementary Table A2, and we report below the corresponding average marginal effects (see section 3.4) because interaction coefficients in nonlinear probability models cannot be interpreted directly as changes in probability.

**Table 3:** Summary probit results—personality heterogeneity.

Dependent variable: intention to adopt a flexitarian diet			
	Model (1)	Model (2)	Model (3)
Benefit framings: all estimates reported			
Financial	−0.176 (0.210)	0.021 (0.228)	0.098 (0.261)
Health	0.191 (0.205)	0.496* (0.218)	0.651** (0.241)
Environmental	0.193 (0.208)	0.324 (0.232)	0.401 (0.261)
Other statistically significant estimates			
Age 35–44	–	n.s.	0.476* (0.231)
Income 50 K-74,999	–	−0.480* (0.243)	−0.504* (0.252)
Environmental morale	–	0.505*** (0.075)	0.538*** (0.081)
Risk attitudes	–	0.109** (0.039)	0.136** (0.042)
Conscientiousness	–	n.s.	0.381* (0.181)
Openness	–	n.s.	0.537* (0.210)
Financial#Extraversion	–	–	−0.534* (0.253)
Health#Conscientiousness	–	–	−0.600** (0.232)
Health#Openness	–	–	−0.705** (0.257)
Environmental#Openness	–	–	−0.581* (0.276)
Model specification			
Socio-demographic controls	No	Yes	Yes
Attitudinal controls	No	Yes	Yes
Personality traits	No	Yes	Yes
Treatment#personality traits	No	No	Yes
*N*	303	303	303
Pseudo *R*^2^	0.011	0.199	0.272
Linktest _hatsq (*p*-value)	–	0.156	0.772
Ramsey Reset test χ^2^ (*p*-value)	–	0.193	0.380

* *p* < 0.05; ** *p* < 0.01; *** *p* < 0.001; robust standard errors reported in parentheses. All benefit-framing estimates are shown irrespective of statistical significance; for brevity, the remaining rows report only statistically significant estimates discussed in the text. “n.s.” indicates a non-significant estimate, while “–” indicates that the variable was not included in the specification. Complete results are reported in Supplementary Table A1.

### 2.5 Statistical analysis

All statistical analyses were conducted using Stata. The probit specifications described above were estimated with robust standard errors, and statistical significance was assessed at the 5% level (*p* < 0.05), with *p* < 0.01 and *p* < 0.001 also reported. Hypotheses were considered supported when the relevant estimated association or treatment effect was statistically significant and, where a directional prediction was specified, consistent with the hypothesised direction. Where only some of the predicted relationships were supported, the hypothesis was described as partially supported. For hypotheses concerning heterogeneous treatment effects, inference was based on the relevant interaction specifications and conditional average marginal effects.

Model adequacy was assessed using variance inflation factors (VIFs), the link test and the Ramsey RESET test. VIF values above 5 were treated as a conservative indication of potentially elevated collinearity, while values above 10 were considered indicative of more serious multicollinearity concerns. For the link test, a non-significant squared predicted-value term, and for the RESET test, a non-significant test of the additional nonlinear terms (*p* > 0.05), were interpreted as providing no evidence of model misspecification. Robustness was further assessed using OLS and ordered probit models retaining the original five-category behavioural-intention measure (see Supplementary Tables A3, A4).

Because several individual-level measures were collected through self-report within the same survey, potential common method bias was assessed using Harman’s single-factor test. An unrotated factor analysis was conducted, with a single factor accounting for more than 50% of the total variance considered indicative of a potential common method concern.

## 3 Results

### 3.1 Empirical specification and interpretation

Our main objective is to examine whether respondents express a positive intention to adopt a flexitarian diet and whether this likelihood varies across benefit-framing conditions and individual characteristics. Accordingly, we code the outcome as a binary indicator and begin our analysis by estimating the following probit model:


$$Y_{i}^{*}=\alpha +\beta^{\prime }T_{i}+\gamma^{\prime }X_{i}+\delta^{\prime }Z_{i}+\theta^{\prime }P_{i}+\lambda^{\prime }(T_{i}\times P_{i})+\varepsilon_{i}$$ (1)

Where the observed outcome is given by:


$$Y_{i}=\{\begin{array}{cc} 1 & \text{if}\;Y_{i}^{*}>0\\ 0 & \text{otherwise} \end{array}$$

and, $Y_{i}^{*}$ denotes the latent propensity of each participant i to express a positive adoption intention, and $Y_{i}$ is the observed binary outcome. $T_{i}$ is a vector of treatment conditions (financial, health, and environmental framings), with the control condition as the reference category. $X_{i}$ is a vector of socio-demographic characteristics (age, gender, education, and income), $Z_{i}$ captures attitudinal variables (environmental morale, risk attitudes, and cognitive reflection), and $P_{i}$ represents personality traits. The interaction term $\left(T_{i}\times P_{i}\right)$ allows the effect of benefit framing to vary across personality traits and $\varepsilon_{i}$ is the error term.

To examine demographic heterogeneity, we re-estimate modified versions of the baseline specification in Equation 1, separately for household income and age. In both models, the personality-trait main effects, $\theta^{\prime }P_{i}$ , the socio-demographic controls contained in $X_{i}$ , and the attitudinal variables contained in $Z_{i}$ are retained. However, the treatment-by-personality interaction term, $\lambda^{\prime }(T_{i}\times P_{i})$ , is replaced by the relevant treatment-by-demographic interaction terms. Accordingly, the income-heterogeneity specification is:


$$Y_{i}^{*}=\alpha +\beta^{\prime }T_{i}+\gamma^{\prime }X_{i}+\delta^{\prime }Z_{i}+\theta^{\prime }P_{i}+\kappa^{\prime }(T_{i}\times I_{i})+\varepsilon_{i}$$ (2)

Where $I_{i}$ denotes the vector of household-income categories. The age-heterogeneity specification is:


$$Y_{i}^{*}=\alpha +\beta^{\prime }T_{i}+\gamma^{\prime }X_{i}+\delta^{\prime }Z_{i}+\theta^{\prime }P_{i}+\psi^{\prime }(T_{i}\times G_{i})+\varepsilon_{i}$$ (3)

Where $G_{i}\;$ denotes the vector of age-group categories. Because the corresponding income and age main effects are already included among the socio-demographic controls in $X_{i}$ , they are not entered separately in the equations. Equations (2, 3) are estimated independently to examine each source of demographic heterogeneity while avoiding the inclusion of a large number of interaction terms in a single model.

In probit regressions, estimated coefficients are not directly interpretable in terms of changes in probability. Rather, their sign indicates whether a variable increases or decreases the latent propensity to switch to a flexitarian diet. To facilitate interpretation, we report average marginal effects from the full model (see model 3 in Table 3) in Figure 2. For continuous variables, marginal effects represent the change in the predicted probability of expressing a positive intention to adopt a flexitarian diet associated with a one-unit increase in the covariate, holding other variables constant; for standardised personality traits, this corresponds to a one standard deviation increase. For binary variables, including treatment indicators, marginal effects capture the discrete change in predicted probability when the variable shifts from 0 to 1 relative to the reference category.

**Figure 2:**
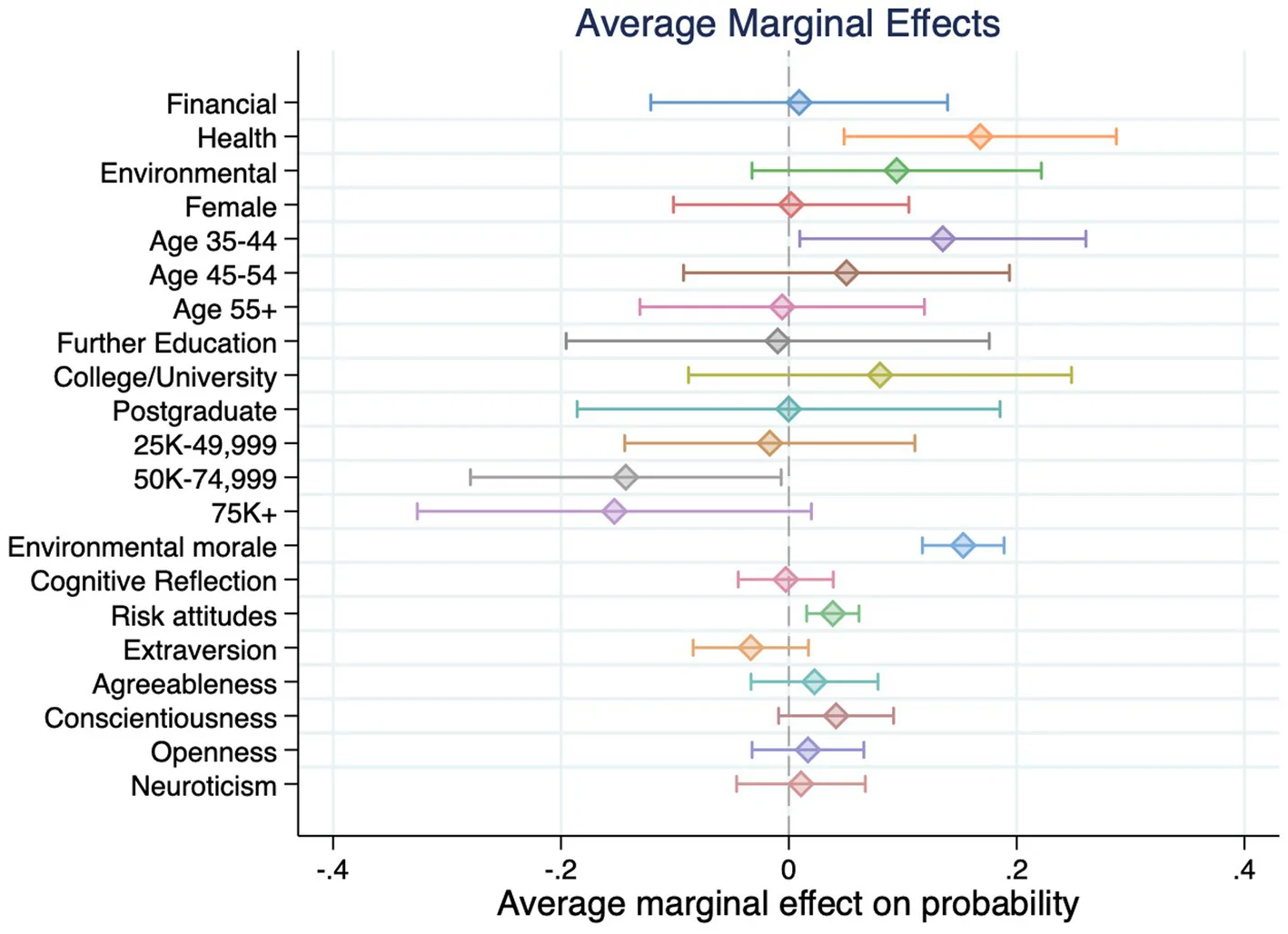
Average marginal effects -personality traits.

Given the non-linear nature of the probit model, interaction coefficients cannot be directly interpreted in probability terms. We therefore compute marginal effects to examine how the relationship between personality traits and behavioural intentions to adopt a flexitarian diet varies across benefit framing conditions. Specifically, we compute marginal effects of personality traits by treatment condition from the fully specified probit model (see model 3 in Table 3), evaluating these effects at each framing condition while holding other covariates at their observed values (see Figure 3).

**Figure 3:**
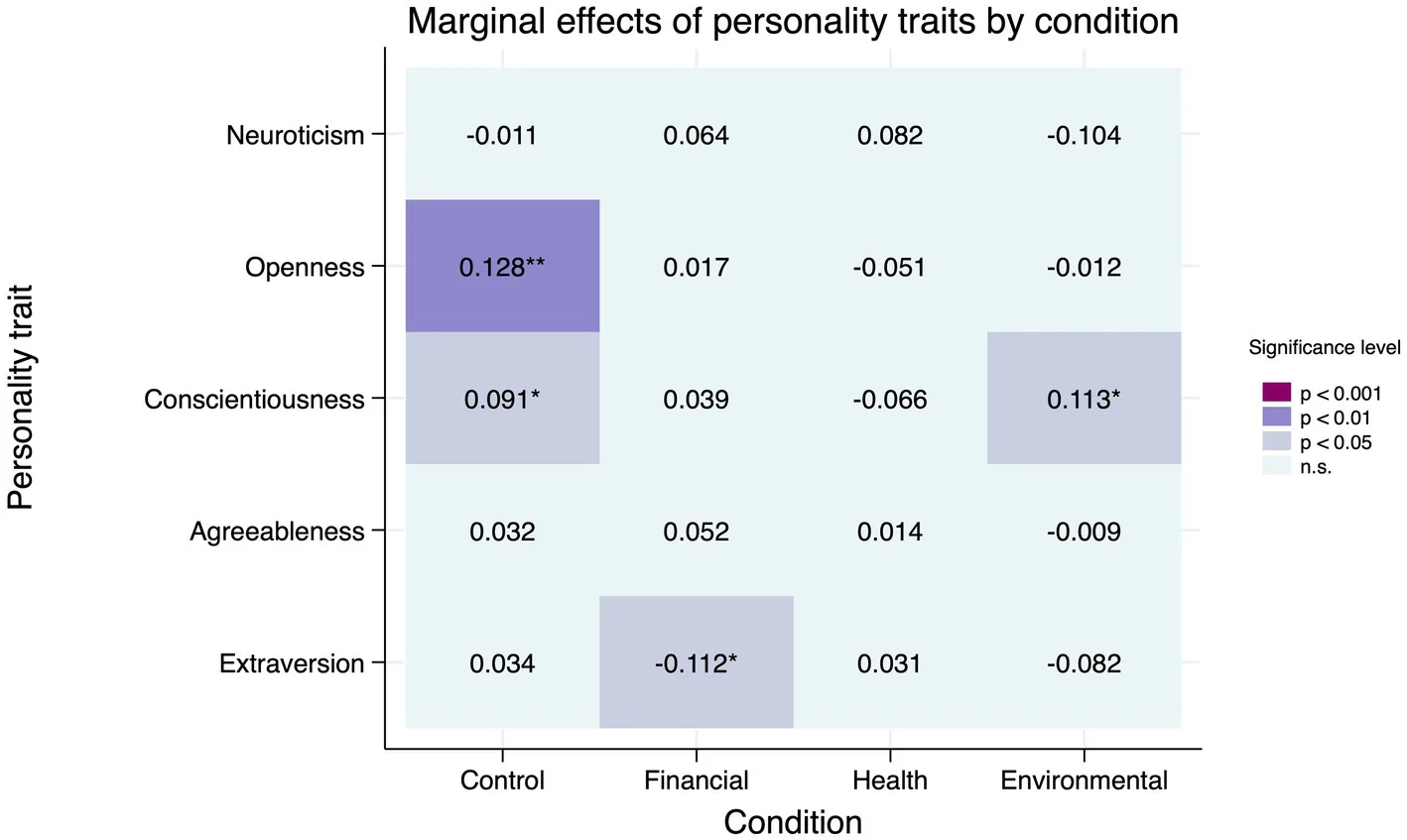
Average marginal effects of personality traits by condition.

Table 3 provides a concise summary of the coefficient estimates highlighted in the discussion and should be read alongside Figures 2, 3, which report the corresponding marginal effects. The estimates presented in Table 3 are drawn from three probit specifications. Model 1 includes the treatment indicators only; Model 2 adds socio-demographic and attitudinal controls; and Model 3 further incorporates personality traits and their interactions with the treatment conditions. The complete regression results, including all coefficient estimates and robust standard errors, are reported in Supplementary Table A1.

### 3.2 Benefit framing and behavioural intention: H1

The results reported in Table 3 highlight the central role of behavioural and attitudinal factors in shaping willingness to adopt a flexitarian diet. In particular, the main effects of the treatment conditions are not statistically significant in specifications excluding socio-demographic, attitudinal, and personality controls (model 1). This pattern changes once individuals’ characteristics are incorporated into the analysis (models 2 and 3). In fact, our analysis reveals that, compared to the control group, being exposed to the health framing has a positive and statistically significant effect ( β =0.496, *p* < 0.05, model 2; β = 0.651, *p* < 0.01, model 3), with average marginal effects suggesting a sizeable increase in the probability of expressing a positive adoption intention (by approximately 17 percentage points, Figure 2). However, while environmental and financial framing also show a positive effect (increasing the intention to adopt a flexitarian diet by approximately 9.5 and 0.9 percentage points), they appear smaller and not statistically significant. These results provide partial support for H1: health framing increases the probability of expressing a positive adoption intention in the fully specified model, whereas financial and environmental framing are not statistically distinguishable from the control condition. Turning to the remaining covariates, among socio-demographic factors, individuals aged 35–44 have an approximately 13.5 percentage-point higher predicted probability of expressing a positive adoption intention than respondents aged 18–34, and this difference is statistically significant at the 5% level (Figure 2; β = 0.476, *p* < 0.05, model 3). Individuals with household incomes between £50 K and £74,999 had a 14.3 percentage-point lower predicted probability of expressing a positive adoption intention than those with household incomes below £25 K (Figure 2). This difference is statistically significant at the 5% level ( β ranges from −0.480 to −0.504, *p* < 0.05; models 2–3).^6^ Looking at the attitudinal variables, environmental morale is consistently and positively associated with the intention to adopt a flexitarian diet and is highly statistically significant ( β ranges from 0.505 to 0.538, *p* < 0.001; models 2–3). A one-unit increase in environmental morale is associated with a 15.3-percentage-point increase in the predicted probability of expressing a positive adoption intention, making it one of the strongest predictors in the models. Risk attitudes are also positively associated with adoption intention and are statistically significant ( β ranges from 0.109 to 0.136, *p* < 0.01; models 2–3), although the corresponding marginal effect is smaller. A one-unit increase in willingness to take risks is only associated with a 3.8-percentage-point increase in the predicted probability of expressing a positive adoption intention (Figure 2).

### 3.3 Personality traits and framing heterogeneity: H2–H5

The inclusion of personality traits reveals substantial heterogeneity in adoption intentions, particularly when the marginal effects are examined separately by framing condition (Figure 3). In the fully specified probit model, the coefficients for conscientiousness ( β = 0.381, *p* < 0.05, model 3, Table 3) and openness ( β = 0.537, *p* < 0.05, model 3, Table 3) are positive in the control condition, providing partial support for H2. The corresponding average marginal effects indicate that a one-standard-deviation increase in openness and conscientiousness is associated with 12.8 and 9.1 percentage-point higher probabilities of expressing a positive adoption intention in the control condition, respectively. These associations, however, vary across framing conditions (Figure 3). The positive association between openness and adoption intention is no longer statistically significant under financial, health, or environmental framing, with negative point estimates under the health and environmental conditions. For conscientiousness, the positive association is attenuated under financial framing and becomes negative but not statistically significant under health framing, contrary to H5. By contrast, it remains positive and statistically significant under environmental framing, where a one-standard-deviation increase in conscientiousness is associated with an 11.3 percentage-point higher probability of expressing a positive intention (*p* < 0.05, Figure 3).

Extraversion also exhibits condition-specific heterogeneity. Under financial framing, a one-standard-deviation increase in extraversion is associated with an 11.2 percentage-point lower probability of expressing a positive adoption intention (*p* < 0.05, Figure 3), consistent with the negative interaction term in Table 3 ( β = − 0.534, *p* < 0.05, model 3).^7^ This suggests that financially framed messages may be less effective among more extraverted individuals. Agreeableness and neuroticism show no consistent or statistically significant associations across conditions. The results therefore provide partial support for H2, but no support for H3 or H4. They are also contrary to H5, as health framing attenuates rather than strengthens the positive association between conscientiousness and adoption intention.

To determine whether the attenuation of the openness and conscientiousness associations reflects lower intentions among individuals higher in these traits or stronger framing effects among those lower in them, we estimated conditional average marginal effects relative to the control condition at one standard deviation below the mean, at the mean, and one standard deviation above the mean of each trait. The corresponding marginal effects are reported in Table 4. Predicted probabilities of expressing a positive adoption intention across framing conditions are presented for openness and conscientiousness in Supplementary Figures A1, A2, respectively. At one standard deviation below the mean of openness, health and environmental framing increase the predicted probability of expressing a positive adoption intention by 36.5 percentage points (*p* < 0.001) and 25.5 percentage points (*p* < 0.01), respectively. At mean openness, health framing remains significant, increasing the probability by 18.5 percentage points (*p* < 0.01), whereas none of the framing effects is statistically significant at one standard deviation above the mean. A similar pattern is observed for conscientiousness. Health framing increases the predicted probability of a positive intention by 32.9 percentage points at one standard deviation below the mean (*p* < 0.001) and by 17.2 percentage points at the mean (*p* < 0.01), but has no statistically significant effect at one standard deviation above the mean. Environmental framing does not differ significantly from the control condition at any of the selected levels of conscientiousness, despite the positive association between conscientiousness and intention within the environmental condition.

**Table 4:** Conditional average marginal effects of benefit framing by openness and conscientiousness.

Personality trait and level	Financial	Health	Environmental
Openness: −1 SD	0.139	0.365***	0.255**
	(0.108)	(0.093)	(0.098)
Openness: mean	0.026	0.185**	0.113
	(0.072)	(0.066)	(0.069)
Openness: +1 SD	−0.104	−0.013	−0.047
	(0.089)	(0.089)	(0.094)
Conscientiousness: −1 SD	0.063	0.329***	0.069
	(0.094)	(0.087)	(0.094)
Conscientiousness: mean	0.010	0.172**	0.096
	(0.068)	(0.063)	(0.069)
Conscientiousness: +1 SD	−0.047	0.007	0.118
	(0.100)	(0.089)	(0.107)

The table reports conditional average marginal effects relative to the control condition. Delta-method robust standard errors are reported in parentheses. Personality traits are standardised; −1 SD, mean, and +1 SD denote one standard deviation below the mean, the mean, and one standard deviation above the mean, respectively. * *p* < 0.05; ** *p* < 0.01; *** *p* < 0.001.

These results indicate that the attenuation of the openness and conscientiousness associations is primarily driven by stronger framing effects among individuals lower in the relevant traits, rather than by statistically significant reductions in intentions among individuals higher in them. The pattern is therefore more consistent with a ceiling effect, whereby individuals higher in openness or conscientiousness already exhibit relatively high intentions in the control condition and consequently have less scope for further increases, than with motivational crowding out or psychological reactance.

### 3.4 Socio-demographic heterogeneity: H6 and H7

To examine socio-demographic heterogeneity and test H6 and H7, we estimate two additional probit models separately from the treatment-by-personality specification reported in Table 3. The first model includes treatment-by-income interactions alongside the treatment indicators and control variables, whereas the second includes treatment-by-age interactions alongside the same set of treatment indicators and controls. Full regression results for these models are provided in Supplementary Table A2. Because interaction coefficients in nonlinear models cannot be interpreted directly as changes in probability, we present the corresponding average marginal effects graphically. Figure 4 reports the average marginal effect of financial framing within each income category, thereby testing whether financial framing is more effective among lower-income respondents (H6).

**Figure 4:**
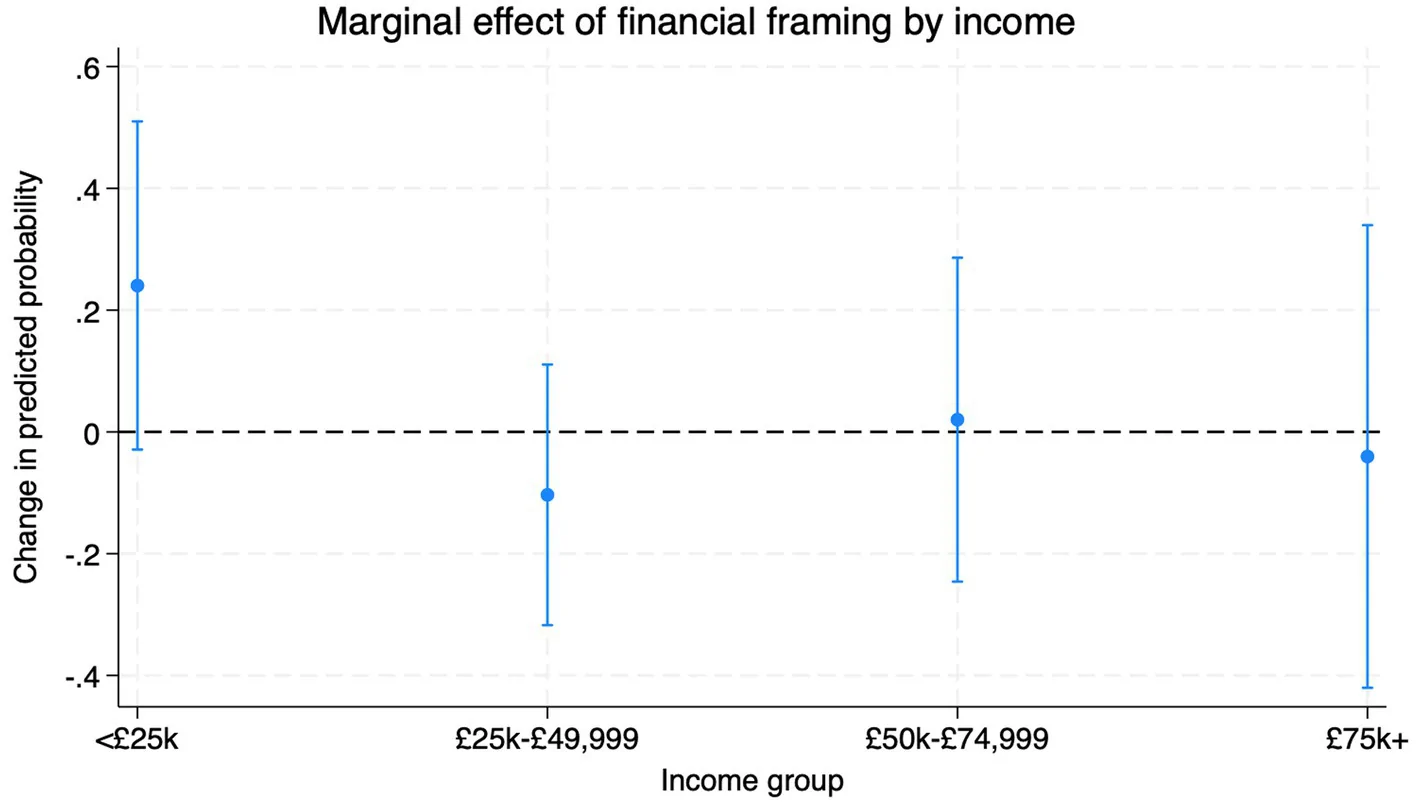
Average marginal effect of financial framing by income.

Figure 5 reports the average marginal effect of health framing within each age category, thereby testing whether health framing is more effective among older respondents (H7).

**Figure 5:**
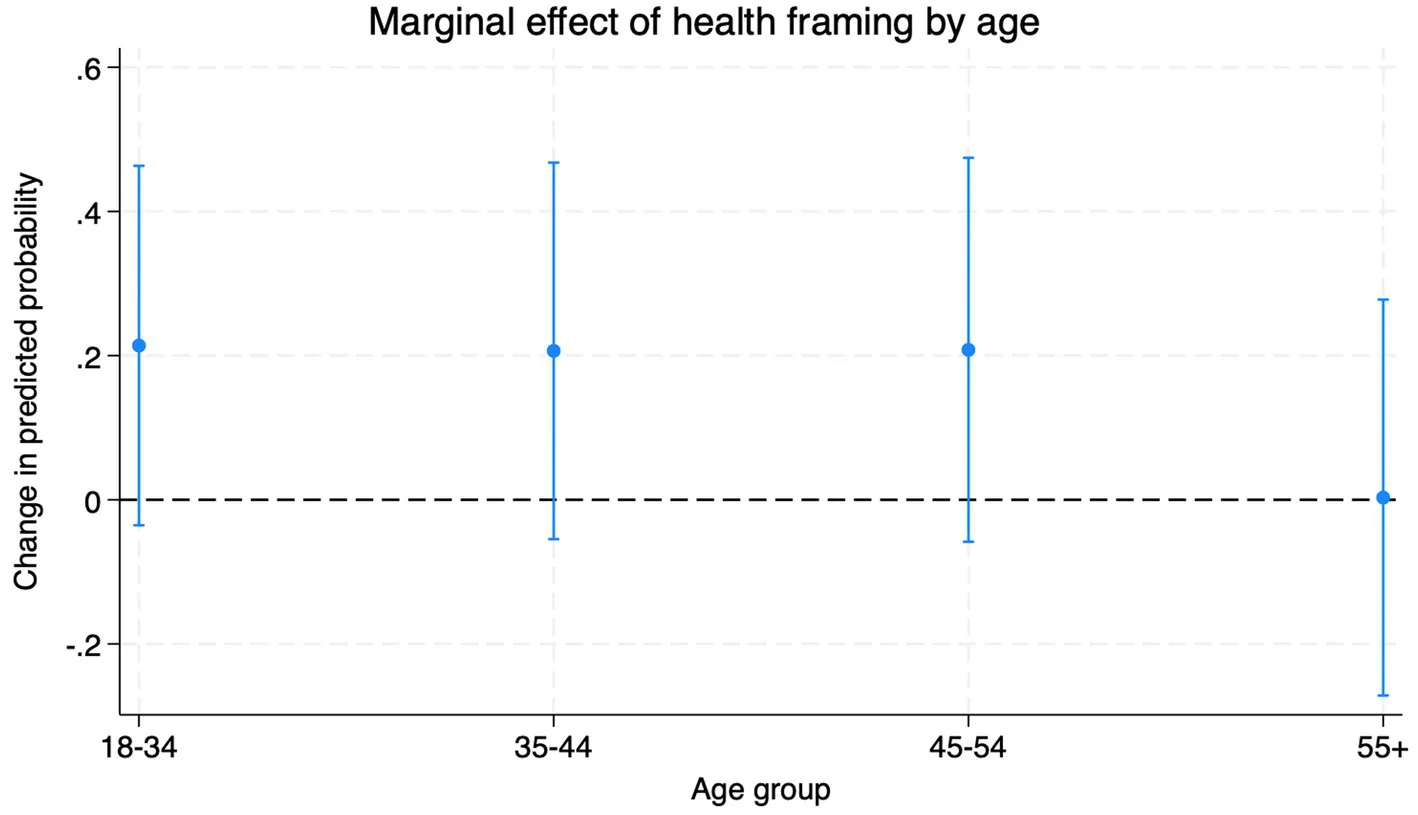
Average marginal effect of health framing by age.

The results provide no support for either hypothesis. Contrary to expectations (H6), financial framing does not appear to be more effective among lower-income individuals. While participants in the lowest-income group (household income below £25 K) show an approximately 24-percentage-point higher intention to adopt a flexitarian diet following financial framing relative to the control condition, this difference is not statistically significant. Similarly, health framing is associated with an increase in the probability of expressing a positive adoption intention among individuals aged 18–54 (approximately 20–25 percentage points), but these effects are not statistically significant and become negligible for individuals aged 55+, providing no support for H7. Thus, although socio-demographic characteristics are associated with baseline behavioural intentions, they do not seem to systematically shape the effectiveness of benefit framing. This highlights the importance of distinguishing between general behavioural tendencies and differential responsiveness to interventions. Nevertheless, some treatment-by-demographic cells inevitably contain fewer observations, which may reduce the precision of the corresponding heterogeneity estimates. These results should therefore be interpreted cautiously.

### 3.5 Robustness checks and limitations

We conducted diagnostic checks to assess the suitability of the regression specifications. Multicollinearity was examined by calculating variance inflation factors from auxiliary OLS regressions containing the same explanatory variables and interaction terms as the corresponding probit models.^8^ The resulting VIF values provide no evidence of problematic multicollinearity. We report the summary VIF results in Table 5. The model containing the treatment indicators, controls and personality traits had a mean VIF of 1.59 and a maximum VIF of 2.82. The treatment-by-personality model had a mean VIF of 2.62 and a maximum of 5.92, while the treatment-by-income and treatment-by-age models had mean VIFs of 3.53 and 3.16 and maximum VIFs of 7.60 and 6.81, respectively. Although some values exceeded the conservative benchmark of 5 in the interaction specifications, none exceeded 10. The higher values were concentrated among variables involved in the interaction terms, for which some increase in collinearity is expected.

**Table 5:** Multicollinearity diagnostics.

Model specification	Mean VIF	Maximum VIF	Variable with maximum VIF
Treatments, controls and personality traits	1.59	2.82	College/university education
Treatment-by-personality	2.62	5.92	Openness
Treatment-by-income	3.53	7.6	£25 K–£49 K income category
Treatment-by-age	3.16	6.81	Age 55+

VIF, variance inflation factor.

Potential common method bias was evaluated using Harman’s single-factor test. The assessment was conducted using the item-level self-reported measures comprising the ten BFI-10 personality items, the two environmental-morale items, the general risk-attitude item, and the original five-category behavioural-intention item. Experimentally assigned treatment conditions, socio-demographic characteristics and the cognitive reflection test were not included, as the assessment focused on measures potentially susceptible to shared response tendencies associated with self-report. The first unrotated factor accounted for 23.7% of the total variance, substantially below the 50% threshold. This provides no indication that the self-reported measures are dominated by a single common response pattern and suggests that common method bias is unlikely to represent a major concern in the present analysis.

All main probit model specifications were also assessed using the link test and the Ramsey RESET test. In each case, the squared predicted-value term in the link test and the additional nonlinear terms in the RESET test were statistically insignificant (i.e., Prob > *χ*^2^ and Prob > |*z*| are greater than 0.1 in all model specifications), providing no evidence of model misspecification or omitted nonlinearities (see Table 3; Supplementary Table A2). Robust standard errors are reported throughout.

Furthermore, to investigate whether the findings depend on the binary coding of behavioural intention, we re-estimated the models in Table 3 and Supplementary Table A1 using an ordered probit specification retaining the original five-category behavioural-intention measure and OLS treating the same five-point outcome as a continuous variable. Results are reported in Supplementary Tables A3, A4.

The principal findings from the fully specified models are broadly consistent across alternative treatments of the outcome. Health framing remains positive and statistically significant in the fully specified probit, OLS, and ordered probit models, whereas the effects of financial and environmental framing are not statistically significant. Environmental morale and greater willingness to take risks also remain positive and statistically significant predictors of behavioural intention across these specifications.

The socio-demographic findings are less consistent. The positive association observed for respondents aged 35–44 in the binary probit model is not statistically significant in the alternative specifications. The negative relationship between higher household income and adoption intention remains broadly evident, although the income category reaching statistical significance varies across models.

Some of the principal personality-related findings are reproduced across specifications. The negative interactions between financial framing and extraversion and between health framing and conscientiousness remain statistically significant in both alternative specifications. By contrast, the interactions between openness and the health and environmental framings are not statistically significant when the original five-category outcome is retained.

Overall, the alternative specifications support the main conclusions regarding treatment effects and attitudinal predictors obtained from the fully specified probit model. The evidence of personality-related heterogeneity is less uniform. The negative interactions involving financial framing and extraversion and health framing and conscientiousness are reproduced across specifications, whereas the openness interactions are specific to the binary probit model. This difference may reflect the distinct dimensions of behavioural intention captured by the models: the binary probit examines the probability of crossing the threshold into a positive adoption intention, while the OLS and ordered probit models examine variation across the full five-category response scale. We therefore place greater emphasis on interaction patterns reproduced across specifications and interpret the openness findings as specific to the fully specified probit model. Future research should assess the stability of these heterogeneous treatment effects using larger samples and observed behavioural outcomes.

Table 6 summarises the results of the hypothesis testing, indicating whether each of the seven hypotheses is supported, partially supported, or not supported based on the empirical findings reported above.

**Table 6:** Summary of hypothesis testing results.

Hypothesis	Empirical finding	Conclusion
H1: Benefit framing → intention	Health: positive, significant; Financial/environmental: n.s.	Partially supported
H2: Big Five → intention	Openness and conscientiousness significant in control condition; other traits not consistently significant	Partially supported
H3: Financial × lower agreeableness	No significant evidence of predicted relationship	Not supported
H4: Environmental × higher agreeableness	No significant evidence of predicted relationship	Not supported
H5: Health × higher conscientiousness	Predicted relationship not observed; effect stronger at lower conscientiousness	Not supported
H6: Financial × lower income	No significant differences in framing effect across income groups	Not supported
H7: Health × older age	No evidence of stronger effect among older respondents	Not supported

n.s., non-significant.

## 4 Discussion of results and concluding remarks

This paper contributes to the literature by examining how three benefit framings (i.e., health, financial, and environmental) influence willingness to adopt a flexitarian diet, while accounting for heterogeneity in personality traits and other individual-level attitudinal and socio-demographic characteristics. Our findings have implications for the promotion of sustainable food systems. Environmental morale and greater willingness to take risks are consistently positively and significantly associated with adoption intention, while the positive effect of health framing is robust across the fully specified probit model and the alternative OLS and ordered probit specifications. However, the estimated interaction patterns suggest that the associations between personality traits and adoption intentions may differ across framing conditions. This indicates that “one-size-fits-all” communication strategies may not be equally relevant or effective across all groups. Future research could therefore examine whether different framings are more relevant to particular groups and whether behaviourally informed strategies that account for psychological heterogeneity improve intervention effectiveness. The effectiveness of such differentiated approaches nevertheless requires direct testing.

The findings are also consistent with a growing body of research in behavioural science and sustainable food consumption. First, the strong and robust association between environmental morale and adoption intention aligns with recent evidence showing that pro-environmental values and attitudes are important predictors of sustainable dietary choices (15, 57, 58). The consistently positive association between greater willingness to take risks and adoption intention further suggests that individual preferences beyond environmental attitudes may also be relevant to individuals’ willingness to consider dietary change. In line with these considerations, recent applications of behavioural frameworks to food systems highlight the central role of attitudes and perceived behavioural control in shaping intentions to adopt sustainable diets (59). This suggests that interventions may be more relevant when they reflect individuals’ underlying attitudes and motivations, rather than relying solely on generic information provision. Similarly, the positive effect of health framing is consistent with recent experimental and applied evidence showing that information-based messages and other behavioural interventions, including changes to the choice environment, can influence food-related intentions and choices, particularly in controlled settings such as cafeterias and restaurants (22, 60, 61).

However, our results also contribute to an increasingly important area of the literature: the heterogeneity of behavioural responses. While many studies report positive average effects of nudges and benefit framing, recent meta-analyses and systematic reviews show that their effectiveness is often modest and highly context-dependent, with outcomes varying across individuals and settings [e.g., (62–64)]. In line with this literature, our results suggest that some personality-related associations vary across framing conditions. Openness and conscientiousness are positively associated with adoption intention in the control condition, but these associations are attenuated under some framing conditions in the fully specified probit model. The negative interactions between financial framing and extraversion and between health framing and conscientiousness are reproduced across the alternative specifications. By contrast, the openness interactions are not statistically significant when the original five-category outcome is retained and should therefore be interpreted more cautiously. Additional conditional marginal-effects analyses indicate that the attenuation of the openness and conscientiousness associations is primarily driven by stronger framing effects among individuals lower in these traits. Health and environmental framing increase intentions among participants lower in openness, while health framing is particularly effective among those lower in conscientiousness. At higher trait levels, these framing effects are not statistically different from the control condition. This pattern is therefore more consistent with a ceiling effect, whereby individuals higher in openness or conscientiousness already express relatively high intentions in the control condition and have less scope for further increases, than with psychological reactance. This interpretation is consistent with critiques of “one-size-fits-all” approaches and supports further investigation of whether differentiated interventions can better account for heterogeneity in preferences and behavioural responses (42).

More broadly, these findings resonate with recent research emphasising that the design and evaluation of interventions may benefit from a better understanding of consumers’ behavioural profiles and food choice motives. Evidence from recent experimental and applied studies in real-world food environments suggests that interventions may be more effective when they are designed around observed behaviours and contextual constraints rather than applied uniformly (64, 65). In this context, our results provide evidence that personality traits are associated with adoption intentions in the control condition and that some of these associations vary across framing conditions. This suggests that evaluations of sustainable-diet interventions may benefit from considering heterogeneity alongside average treatment effects and from examining whether behavioural segmentation could improve message relevance.

One possible direction for future research is to test whether communication informed by environmental attitudes, socio-demographic characteristics, or personality-related measures improves message relevance or effectiveness. Ardebili and Rickertsen (31) discuss similar approaches after identifying associations between personality traits, including openness and conscientiousness, and dietary preferences in survey data. Although personality traits may, to some extent, be inferred from digital behaviour and online footprints (66), the use of such data raises important concerns regarding privacy, consent, transparency, measurement validity, and ethical implementation. More broadly, behavioural insights may also inform communication strategies involving organisations, local authorities, retailers, and other actors that influence food systems and practises (19). However, the effectiveness and scalability of such approaches should be established through direct evaluation rather than inferred from the present study.

Overall, our findings suggest that the design and evaluation of interventions promoting sustainable food systems may benefit from greater attention to behavioural heterogeneity. From a practical perspective, health-focused information may provide a useful starting point for communication strategies aimed at encouraging consideration of flexitarian diets, as health framing was the only message with a robust positive average effect across model specifications. By contrast, financial and environmental benefit messages should not be assumed to increase intentions when used in isolation. The observed heterogeneity further suggests that uniform messaging may overlook differences in responsiveness across individuals. Public-health bodies, local authorities, retailers and other actors promoting sustainable diets could therefore consider testing alternative framings across different audience segments rather than relying on a single communication strategy. Social and dynamic norms may also offer promising complementary approaches, although their effectiveness in more targeted settings remains to be established (67). More differentiated strategies could also involve adapting communication approaches or choice environments through measures such as defaults or product placement. However, the effectiveness of such targeted and context-sensitive approaches should be evaluated directly using larger samples and observed dietary behaviour before firm policy conclusions are drawn.

## Data Availability

The original contributions generated in this paper are included in the main manuscript and Supplementary material. Further inquiries can be directed to the corresponding author.

## Footnotes

1 These include defaults, product placement, and change in availability to information and messaging-based approaches that communicate particular benefits or consequences.

2 Ethical approval for this study was granted by the University of Warwick (reference: BES_BDS_2025_029).

3 Although the realised sample size was sufficient to examine medium-sized pairwise differences on the original five-point outcome, the study was not designed specifically to detect small treatment-by-personality or treatment-by-demographic interaction effects; these heterogeneity analyses therefore have lower precision than the main treatment comparisons.

4 As no discrepancies were observed between the variables gender and sex, we use the gender variable to capture gender differences.

5 Each personality domain included one reversed statement per domain (see Supplementary Appendix A).

6 This pattern is consistent with higher income brackets, though the effect is not statistically significant at 5% significance level.

7 Figure 3 shows that extraversion is not significantly associated with adoption intention in the control condition but is negatively associated with intention under financial framing. The significant Financial#Extraversion interaction term in Table 3, model 3, formally indicates that these condition-specific associations differ. It does not imply that financial framing has a statistically significant average effect across participants.

8 Variance inflation factors cannot be obtained directly following probit estimation in Stata. Full OLS regression results and supplementary multicollinearity diagnostics are available from the author upon request.
