# Supplementary material for: Benefit framing, personality traits and intention to adopt a flexitarian diet
Source: Front Nutr. 2026 Oct 15;13:1848757. doi: 10.3389/fnut.2026.1848757 (PMC13718577; doi:10.3389/fnut.2026.1848757)
Supplement: Supplementary file 1 [file Supplementary_file_1.docx]

Supplementary Material

**Appendix A**

**Survey given to Participants on Prolific**

**BFI – 10:**

I see myself as someone who…

… is reserved (rev.)

… is outgoing, sociable

… is generally trusting

… tends to find fault with others (rev.)

… does a thorough job

… tends to be lazy (rev.)

… is relaxed, handles stress well (rev.)

… gets nervous easily

… has an active imagination

… has few artistic interests (rev.)

Answers:

1 = Strongly disagree, 2 = Disagree, 3 = Undecided/I cannot say, 4 = Agree, 5 = Strongly agree

**Instructional Manipulation Check 1:**

Most modern theories of decision making recognise that decisions do not take place in a vacuum. Individual preferences and knowledge, along with situational variables can greatly impact the decision process. To demonstrate that you’ve read this much please select both yellow and blue among the alternatives below, no matter what your favourite colour is. Yes,

ignore the question below and select both of those option. “What is your favorite color?”

Yellow, Blue, Green, Purple, Orange, I prefer not to say

**Behavioural intention to Adopt a Flexitarian Diet:**

Definition (all conditions):

Switching to a Flexitarian Diet Per a University of Oxford study’s definition a flexitarian diet is one that includes at least 500g per day of fruits and vegetables of different colours and groups (the composition of which is determined by regional preferences), at least 100g per day of plant-based protein sources (legumes, soybeans, nuts), modest amounts of animal-based proteins (e.g. less than two servings per week of each poultry, fish, and eggs, and less than one serving per day of milk or dairy), and limited amounts of red meat (1 portion per week or less), processed sugar (candies, cookies), vegetable oils that are high in saturated fat (in particular palm oil), and starchy foods which have a relatively high glycaemic index.

Financial Benefit Info (added to financial condition):

The same study estimated that if residents in high-income countries like the UK take on an energy-balanced flexitarian diet as opposed to the current average diet they could save approximately 14% of the money they spend, buying their groceries. A dietary switch like this would save someone living in the UK approximately £273 per person per year.

Monetary savings calculated as follows (not presented to participants):

Benefit information was calculated by taking 7.09$ USD, the benchmark cost of a benchmark diet per day in high-income countries (HICs), and reducing it by 14%, according to the study in HICs for a FLX diet (see Springmann et al., 2021). That was equal to 6.10$ USD. The difference between the two diets is 361.35$ USD, or approximately 273 GBP per year, which was calculated by multiplying the diets’ daily costs by 365.

Health Benefit Info (added to health condition):

The same researchers in a different study estimated that if residents in high-income countries like the UK take on an energy-balanced flexitarian diet as opposed to the current average diet they could reduce their risk of premature mortality by approximately 20% by reducing the risk of coronary heart disease, stroke, type 2 diabetes, all various cancer types, as well as an aggregate of causes that are associated with changes in weight. In other words, about 1 in 5 passings that would otherwise occur due to diet-related chronic disease can be prevented by switching from the current average UK diet to a flexitarian diet.

Environmental Benefit info (added to environmental condition):

The same researchers in a different study estimated that if residents in high-income countries like the UK take on an energy-balanced flexitarian diet as opposed to the current average diet they could reduce greenhouse gas emissions that their diet accounts for by approximately 75%. A dietary switch like this would prevent the emission of approximately 996kg of CO2 per person over the course of a year. That is roughly equivalent to the yearly CO2 absorption of 100 trees (1 urban medium-sized tree absorbs approximately 10 kg of CO2 over a year).

Environmental benefits calculated as follows (not presented to participants):

The supplementary tables from Springmann et al. (2018) were evaluated for this benefit information. The HIC benchmark diet of 2030 food amounts in Supplementary Table 3 that give an estimate of how many grams of each food group the average HIC diet in 2030 would consist of was multiplied by the GHG intensity using kgCO2/kg data on those same food groups from Supplementary Table 10. The same was done for the flexitarian diet using Supplementary Table 4 and 10. This gives an estimate of the GHG emissions for each diet per day on average. Benchmark HIC: 3.640 kg CO₂-eq/day; Flexitarian: 0.911 kg CO₂-eq/day. Their difference was calculated: 2.729 kg CO₂-eq/day and then multiplied by 365 for a yearly estimate: approximately 996 kg CO₂-eq/year.

Behavioural Intention Statement: (added at the end of the vignette to all conditions)

How strongly would you agree or disagree with the following statement. I would agree to take on a flexitarian diet starting today.

1 = Strongly disagree, 2 = Disagree, 3 = Undecided/I cannot say, 4 = Agree, 5 = Strongly agree

**Demographic Survey Pt. 1**

**About You**

Gender: How do you identify? [choose one only]

Male

Female

Non-binary

Prefer to self-describe [fill in the blank option]

Prefer not to say

Sex: What is your sex, as recorded on legal/official documents?

Male

Female

Prefer not to say

Age: What is your age group? [choose one only]

18 to 24

25 to 34

35 to 44

45 to 54

55 to 64

65 or over

Prefer not to say

Ethnicity: What is your ethnic group? (Choose one option that best describes your ethnic group or background) [choose one only]

Asian/Asian British

a. Indian
b. Pakistani
c. Bangladeshi
d. Chinese
e. Any other Asian background, please describe

Black / African / Caribbean / Black British

a. African
b. Caribbean
c. Any other Black / African / Caribbean background, please describe

Mixed / Multiple ethnic groups

a. White and Black Caribbean
b. White and Black African
c. White and Asian
d. Any other Mixed / Multiple ethnic background, please describe

White

a. English / Welsh / Scottish / Northern Irish / British
b. Irish
c. Gypsy or Irish Traveller
d. Any other White background, please describe

Other ethnic group

a. Arab
b. Any other ethnic group, please describe

Prefer not to say

What is your religion [choose one only]:

Buddhist

Christian

Hindu

Jewish

Muslim

Sikh

Other religion, please describe

No religion

Prefer not to say

Marital status [choose one only]:

Married

Living as a couple

Separated

Divorced

Widowed

Single

Other [fill in the blank option]

Prefer not to say

Number of children: How many children do you have that live at home with you or who you have regular responsibility for? [choose one only]

None

1

2

3

4

5 or more

Prefer not to say

UK Geographical Region: In what region do you reside? (choose one only)

London

North East

North West

Yorkshire and the Humber East Midlands

West Midlands

East of England

South East

South West

Scotland

Wales

Northern Ireland

Prefer not to say

Education: What is the highest level of education you have completed? [choose one only]

Primary school

Secondary school up to 16 years

Higher or secondary or further education (A-levels, BTEC, etc.)

College or university

Post-graduate degree

Prefer not to say

Occupation: What is your current employment status? [choose one only]

Part-time employee

Full-time employee

Part-time self-employed

Full-time self-employed

Not employed

Retired

Student

At home

Carer of another household member

Other [fill in the blank option]

Prefer not to say

Household income: What is your annual household income, which is the total amount that each individual in your home made within the last year? [choose one only]

Less than £10,000

Between £10,000-£24,999

Between £25,000-£49,999

Between £50,000-£74,999

Between £75,000-£99, 999

Between £100,000-£124,999

More than £125,000

Prefer not to say

Which of the following political parties do you most identify with? (choose one only)

Labour

Conservative

Scottish National Party (SNP)

Liberal Democrats

Reform UK

Green Party

UKIP

Plaid Cymru

Jeremy Corbyn & Zarah Sultana's New Party

Other, please describe

Prefer not to say

**Instructional Manipulation Check 2**

We are also interested in what sections people like to read in the newspaper. This might affect what they learn from articles and how they feel about issues discussed in them. We also want to see if people are reading the questions carefully. To show that you've read this much, please mark both the sports and none of the above boxes below. That's right, just select these two options only. Regardless of how frequently you read the newspaper, what would you say are your favourite newspaper sections to read? (please check all that apply)

National

Local

Real Estate

Comics

Classified

Style

Sports

Business

Science and Technology

Opinion

None of the above

All of the above

**Demographic Survey Pt. 2**

Diet: Do you currently follow any of the following diets? (choose one only)

I do not follow any specific diet

Vegetarian Diet

Vegan Diet

Pescatarian Diet

Paleo Diet

Ketogenic Diet

Other, please describe

Prefer not to say

Risk attitudes:

Risk attitudes: Are you generally a person who is fully prepared to take risks or do you try to avoid taking risks? Please use the following scale, where the 0 means “I try to avoid taking risks” and 10 means “I am fully prepared to take risks’.

1, 2, 3, 4, 5, 6, 7, 8, 9, 10

Environmental morale/pro-environmental attitudes [from European Value Survey, EVS, Torgler and Garcia-Valiñas (2007)]:

Environmental attitudes: Please state to what extent do you agree on the following statements:

a) I would give part of my income if I were certain that the money would be used to prevent environmental pollution (1= strongly disagree, 5 = strongly agree)

1, 2, 3, 4, 5

b) I would agree to an increase in taxes if the extra money were used to prevent environmental pollution (1 = strongly disagree, 5 = strongly agree)

1, 2, 3, 4, 5

Cognitive Reflection Test

(1) A bat and a ball cost $1.10 in total. The bat costs $1.00 more than the ball. How much does the ball cost? _____ cents

(2) If it takes 5 machines 5 minutes to make 5 widgets, how long would it take 100 machines to make 100 widgets? _____ minutes

(3) In a lake, there is a patch of lily pads. Every day, the patch doubles in size. If it takes 48 days for the patch to cover the entire lake, how long would it take for the patch to cover half of the lake? _____ days

**End of survey**

| **Table A1: Full Probit Results - Personality Heterogeneity** | | | |  |
| --- | --- | --- | --- | --- |
| Dependent variable: intention to adopt a flexitarian diet | | | |  |
| Variable | (1) | (2) | (3) |  |
| Financial | -0.176 | 0.021 | 0.098 |  |
|  | (0.210) | (0.228) | (0.261) |  |
| Health | 0.191 | 0.496* | 0.651** |  |
|  | (0.205) | (0.218) | (0.241) |  |
| Environmental | 0.193 | 0.324 | 0.401 |  |
|  | (0.208) | (0.232) | (0.261) |  |
| Gender |  | -0.048 | 0.007 |  |
|  |  | (0.175) | (0.185) |  |
| *Age 18-34 (base)* |  |  |  |  |
| Age 35-44 |  | 0.353 | 0.476* |  |
|  |  | (0.214) | (0.231) |  |
| Age 45-54 |  | 0.210 | 0.179 |  |
|  |  | (0.247) | (0.257) |  |
| Age 55+ |  | 0.013 | -0.021 |  |
|  |  | (0.228) | (0.227) |  |
| *Education - Up to secondary (base)* | |  |  |  |
| Further Education |  | 0.086 | -0.035 |  |
|  |  | (0.315) | (0.337) |  |
|  |  |  |  |  |
| College/University |  | 0.319 | 0.282 |  |
|  |  | (0.282) | (0.306) |  |
| Postgraduate |  | 0.203 | -0.001 |  |
|  |  | (0.323) | (0.337) |  |
| *Income <25K (base)* |  |  |  |  |
| 25K-49,999 |  | -0.050 | -0.058 |  |
|  |  | (0.213) | (0.228) |  |
| 50K-74,999 |  | -0.480* | -0.504* |  |
|  |  | (0.243) | (0.252) |  |
| 75K+ |  | -0.447 | -0.540 |  |
|  |  | (0.302) | (0.324) |  |
| Environmental morale |  | 0.505*** | 0.538*** |  |
|  |  | (0.075) | (0.081) |  |
| Cognitive Reflection (Test score) | | -0.004 | -0.009 |  |
|  |  | (0.071) | (0.075) |  |
| Risk Attitudes |  | 0.109** | 0.136** |  |
|  |  | (0.039) | (0.042) |  |
| Extraversion |  | -0.080 | 0.142 |  |
|  |  | (0.091) | (0.181) |  |
| Agreeableness |  | 0.057 | 0.135 |  |
|  |  | (0.094) | (0.201) |  |
| Conscientiousness |  | 0.115 | 0.381* |  |
|  |  | (0.096) | (0.181) |  |
| Openness |  | -0.002 | 0.537* |  |
|  |  | (0.089) | (0.210) |  |
| Neuroticism |  | 0.022 | -0.045 |  |
|  |  | (0.097) | (0.195) |  |
| Financial#Extraversion |  |  | -0.534* |  |
|  |  |  | (0.253) |  |
| Health#Extraversion |  |  | -0.040 |  |
|  |  |  | (0.238) |  |
| Environment#Extraversion |  |  | -0.440 |  |
|  |  |  | (0.284) |  |
| Financial#Agreeableness |  |  | 0.047 |  |
|  |  |  | (0.277) |  |
| Health#Agreeableness |  |  | -0.089 |  |
|  |  |  | (0.270) |  |
| Environmental#Agreeableness | |  | -0.169 |  |
|  |  |  | (0.320) |  |
| Financial#Conscientiousness | |  | -0.244 |  |
|  |  |  | (0.259) |  |
| Health#Conscientiousness |  |  | -0.600** |  |
|  |  |  | (0.232) |  |
| Environment#Conscientiousness | |  | 0.029 |  |
|  |  |  | (0.277) |  |
| Financial#Openness |  |  | -0.478 |  |
|  |  |  | (0.274) |  |
| Health#Openness |  |  | -0.705** |  |
|  |  |  | (0.257) |  |
| Environmental#Openness |  |  | -0.581* |  |
|  |  |  | (0.276) |  |
| Financial#Neuroticism |  |  | 0.267 |  |
|  |  |  | (0.259) |  |
| Health#Neuroticism |  |  | 0.319 |  |
|  |  |  | (0.254) |  |
| Environmental#Neuroticism | |  | -0.333 |  |
|  |  |  | (0.307) |  |
| Constant | -0.160 | -2.497*** | -2.771*** |  |
|  | (0.150) | (0.461) | (0.518) |  |
| N | 303 | 303 | 303 |  |
| Prob > χ² | 0.216 | 0.000 | 0.000 |  |
| Pseudo R² | 0.011 | 0.199 | 0.272 |  |
| Linktest _hat (p-value) |  | 0.000 | 0.000 |  |
| Linktest _hatsq (p-value) |  | 0.156 | 0.772 |  |
| Ramsey Reset test χ² (p-value) | | 0.193 | 0.380 |  |
| *Notes:* *p < 0.05; **p < 0.01; ***p < 0.001; robust standard errors reported in parentheses. (1), (2), and (3) represent respectively Model 1, Model 2 and Model 3. | | | |  |
|  |  |  |  |  |

| **Table A2: Probit Results – Socio-Demographic Heterogeneity** | | | |  |
| --- | --- | --- | --- | --- |
| Dependent variable: intention to adopt a flexitarian diet | | | |  |
| Panel A: Treatment x Income | | Panel B. Treatment × Age | |  |
| Gender | 0.008 | Gender | -0.065 |  |
|  | (0.179) |  | (0.182) |  |
| *Age 18-34 (base)* |  | *Education - Up to secondary (base)* | |  |
| Age 35-44 | 0.373 | Further Education | 0.015 |  |
|  | (0.214) |  | (0.329) |  |
| Age 45-54 | 0.227 | College/University | 0.284 |  |
|  | (0.248) |  | (0.287) |  |
| Age 55+ | 0.110 | Postgraduate | 0.171 |  |
|  | (0.227) |  | (0.326) |  |
| *Education - Up to secondary (base)* | | *Income <25K (base)* |  |  |
| Further Education | 0.117 | 25K-49,999 | -0.079 |  |
|  | (0.327) |  | (0.218) |  |
| College/University | 0.349 | 50K-74,999 | -0.498* |  |
|  | (0.289) |  | (0.251) |  |
| Postgraduate | 0.261 | 75K+ | -0.464 |  |
|  | (0.328) |  | (0.300) |  |
| Environmental morale | 0.531*** | Environmental morale | 0.513*** |  |
|  | (0.079) |  | (0.078) |  |
| Cognitive Reflection (Test score) | -0.018 | Cognitive Reflection (Test score) | -0.013 |  |
|  | (0.073) |  | (0.073) |  |
| Risk Attitudes | 0.122** | Risk Attitudes | 0.114** |  |
|  | (0.040) |  | (0.039) |  |
| Extraversion | -0.067 | Extraversion | -0.074 |  |
|  | (0.091) |  | (0.091) |  |
| Agreeableness | 0.048 | Agreeableness | 0.077 |  |
|  | (0.095) |  | (0.095) |  |
| Conscientiousness | 0.107 | Conscientiousness | 0.124 |  |
|  | (0.103) |  | (0.097) |  |
| Openness | -0.018 | Openness | -0.013 |  |
|  | (0.090) |  | (0.092) |  |
| Neuroticism | 0.028 | Neuroticism | 0.045 |  |
|  | (0.099) |  | (0.100) |  |
| Financial | 0.737 | Financial | -0.166 |  |
|  | (0.470) |  | (0.431) |  |
| Health | 1.025* | Health | 0.663 |  |
|  | (0.460) |  | (0.449) |  |
| Environmental | 0.557 | Environmental | 0.016 |  |
|  | (0.490) |  | (0.429) |  |
| *Income <25K (base)* |  | *Age 18-34 (base)* |  |  |
| 25K-49,999 | 0.429 | Age 35-44 | 0.195 |  |
|  | (0.443) |  | (0.434) |  |
| 50K-74,999 | -0.008 | Age 45-54 | -0.251 |  |
|  | (0.485) |  | (0.481) |  |
| 75K+ | 0.009 | Age 55+ | 0.179 |  |
|  | (0.580) |  | (0.478) |  |
| Financial × £25K-£49,999 | -1.082 | Financial × Age 35-44 | 0.206 |  |
|  | (0.582) |  | (0.591) |  |
| Financial × £50K-£74,999 | -0.649 | Financial × Age 45-54 | 0.987 |  |
|  | (0.642) |  | (0.734) |  |
| Financial × £75K+ | -0.738 | Financial × Age 55+ | -0.230 |  |
|  | (0.840) |  | (0.657) |  |
| Health × £25K-£49,999 | -0.411 | Health × Age 35-44 | -0.074 |  |
|  | (0.590) |  | (0.630) |  |
| Health × £50K-£74,999 | -1.088 | Health × Age 45-54 | 0.173 |  |
|  | (0.647) |  | (0.644) |  |
| Health × £75K+ | -0.608 | Health × Age 55+ | -0.695 |  |
|  | (0.760) |  | (0.650) |  |
| Environmental × £25K-£49,999 | -0.373 | Environmental × Age 35-44 | 0.423 |  |
|  | (0.629) |  | (0.626) |  |
| Environmental × £50K-£74,999 | -0.132 | Environmental × Age 45-54 | 0.679 |  |
|  | (0.656) |  | (0.656) |  |
| Environmental × £75K+ | -0.564 | Environmental × Age 55+ | 0.238 |  |
|  | (0.818) |  | (0.635) |  |
| Constant | -3.070*** | Constant | -2.360*** |  |
|  | (0.583) |  | (0.529) |  |
| N | 303 | N | 303 |  |
| Prob > χ² | 0.000 | Prob > χ² | 0.000 |  |
| Pseudo R² | 0.217 | Pseudo R² | 0.212 |  |
| Linktest _hat (p-value) | 0.000 | Linktest _hat (p-value) | 0.000 |  |
| Linktest _hatsq (p-value) | 0.129 | Linktest _hatsq (p-value) | 0.423 |  |
| Ramsey Reset test χ² (p-value) | 0.2022 | Ramsey Reset test χ² (p-value) | 0.3540 |  |
| Notes: *p < 0.05; **p < 0.01; ***p < 0.001; robust standard errors reported in parentheses. Panel A reports results for the treatment-by-income interactions alongside the treatment indicators and control variables, whereas Panel B includes treatment-by-age interactions alongside the same set of treatment indicators and controls. | | | |  |
|  |  |  |  |  |
|  |  |  |  |  |
|  |  |  |  |  |

| **Table A3: OLS Results - Personality Heterogeneity** | | | |  |
| --- | --- | --- | --- | --- |
| Dependent variable: Strength of intention to adopt a flexitarian diet | | | |  |
| Variable | (1) | (2) | (3) |  |
| Financial | -0.103 | 0.097 | 0.106 |  |
|  | (0.208) | (0.180) | (0.176) |  |
| Health | 0.216 | 0.377** | 0.434*** |  |
|  | (0.199) | (0.166) | (0.165) |  |
| Environmental | 0.186 | 0.278 | 0.274 |  |
|  | (0.215) | (0.194) | (0.190) |  |
| Gender |  | 0.042 | 0.063 |  |
|  |  | (0.142) | (0.139) |  |
| *Age 18-34 (base)* |  |  |  |  |
| Age 35-44 |  | 0.192 | 0.225 |  |
|  |  | (0.173) | (0.170) |  |
| Age 45-54 |  | 0.259 | 0.203 |  |
|  |  | (0.194) | (0.186) |  |
| Age 55+ |  | 0.157 | 0.098 |  |
|  |  | (0.173) | (0.171) |  |
| *Education - Up to secondary (base)* | |  |  |  |
| Further Education |  | 0.102 | 0.113 |  |
|  |  | (0.231) | (0.235) |  |
| College/University |  | 0.207 | 0.247 |  |
|  |  | (0.217) | (0.216) |  |
| Postgraduate |  | 0.274 | 0.208 |  |
|  |  | (0.257) | (0.247) |  |
| *Income <25K (base)* |  |  |  |  |
| 25K-49,999 |  | -0.017 | -0.019 |  |
|  |  | (0.169) | (0.163) |  |
| 50K-74,999 |  | -0.290 | -0.280 |  |
|  |  | (0.195) | (0.189) |  |
| 75K+ |  | -0.438* | -0.457** |  |
|  |  | (0.229) | (0.217) |  |
| Environmental morale |  | 0.381*** | 0.366*** |  |
|  |  | (0.057) | (0.056) |  |
| Cognitive Reflection (Test score) | | -0.092 | -0.098* |  |
|  |  | (0.058) | (0.056) |  |
| Risk Attitudes |  | 0.099*** | 0.099*** |  |
|  |  | (0.030) | (0.030) |  |
| Extraversion |  | -0.102 | 0.028 |  |
|  |  | (0.073) | (0.125) |  |
| Agreeableness |  | 0.124 | 0.147 |  |
|  |  | (0.076) | (0.134) |  |
| Conscientiousness |  | 0.116 | 0.414*** |  |
|  |  | (0.076) | (0.122) |  |
| Openness |  | 0.034 | 0.162 |  |
|  |  | (0.069) | (0.156) |  |
| Neuroticism |  | 0.056 | 0.152 |  |
|  |  | (0.083) | (0.125) |  |
| Financial#Extraversion |  |  | -0.422** |  |
|  |  |  | (0.178) |  |
| Health#Extraversion |  |  | 0.062 |  |
|  |  |  | (0.166) |  |
| Environment#Extraversion |  |  | -0.224 |  |
|  |  |  | (0.212) |  |
| Financial#Agreeableness |  |  | 0.112 |  |
|  |  |  | (0.194) |  |
| Health#Agreeableness |  |  | -0.066 |  |
|  |  |  | (0.186) |  |
| Environmental#Agreeableness | |  | -0.094 |  |
|  |  |  | (0.239) |  |
| Financial#Conscientiousness | |  | -0.398** |  |
|  |  |  | (0.191) |  |
| Health#Conscientiousness |  |  | -0.474*** |  |
|  |  |  | (0.167) |  |
| Environment#Conscientiousness | |  | -0.240 |  |
|  |  |  | (0.210) |  |
| Financial#Openness |  |  | -0.052 |  |
|  |  |  | (0.197) |  |
| Health#Openness |  |  | -0.166 |  |
|  |  |  | (0.191) |  |
| Environmental#Openness |  |  | -0.172 |  |
|  |  |  | (0.201) |  |
| Financial#Neuroticism |  |  | 0.038 |  |
|  |  |  | (0.186) |  |
| Health#Neuroticism |  |  | 0.063 |  |
|  |  |  | (0.167) |  |
| Environmental#Neuroticism | |  | -0.512** |  |
|  |  |  | (0.235) |  |
| Constant | 2.972*** | 1.234*** | 1.255*** |  |
|  | (0.155) | (0.312) | (0.316) |  |
| N | 303 | 303 | 303 |  |
| R² | 0.012 | 0.279 | 0.357 |  |
| Notes: *p < 0.05; **p < 0.01; ***p < 0.001; robust standard errors reported in parentheses. (1), (2), and (3) represent respectively Model 1, Model 2 and Model 3. | | | |  |
|  |  |  |  |  |

| **Table A4: Ordered Probit Results - Personality Heterogeneity** | | | |  |
| --- | --- | --- | --- | --- |
| Dependent variable: Strength of intention to adopt a flexitarian diet | | | |  |
| Variable | (1) | (2) | (3) |  |
| Financial | -0.077 | 0.124 | 0.130 |  |
|  | (0.179) | (0.179) | (0.183) |  |
| Health | 0.158 | 0.351* | 0.437* |  |
|  | (0.170) | (0.169) | (0.175) |  |
| Environmental | 0.133 | 0.253 | 0.280 |  |
|  | (0.189) | (0.199) | (0.207) |  |
| Gender |  | 0.052 | 0.084 |  |
|  |  | (0.142) | (0.142) |  |
| *Age 18-34 (base)* |  |  |  |  |
| Age 35-44 |  | 0.167 | 0.202 |  |
|  |  | (0.172) | (0.177) |  |
| Age 45-54 |  | 0.279 | 0.229 |  |
|  |  | (0.190) | (0.191) |  |
| Age 55+ |  | 0.203 | 0.170 |  |
|  |  | (0.170) | (0.172) |  |
| *Education - Up to secondary (base)* | |  |  |  |
| Further Education |  | 0.041 | 0.081 |  |
|  |  | (0.227) | (0.238) |  |
| College/University |  | 0.210 | 0.294 |  |
|  |  | (0.210) | (0.215) |  |
| Postgraduate |  | 0.288 | 0.263 |  |
|  |  | (0.252) | (0.251) |  |
| *Income <25K (base)* |  |  |  |  |
| 25K-49,999K |  | -0.047 | -0.047 |  |
|  |  | (0.168) | (0.172) |  |
| 50K-74,999K |  | -0.305 | -0.313 |  |
|  |  | (0.197) | (0.200) |  |
| 75K+ |  | -0.474* | -0.531* |  |
|  |  | (0.226) | (0.222) |  |
| Environmental morale |  | 0.381*** | 0.393*** |  |
|  |  | (0.061) | (0.064) |  |
| Cognitive Reflection (Test score) | | -0.104 | -0.118 |  |
|  |  | (0.060) | (0.060) |  |
| Risk Attitudes |  | 0.106*** | 0.112*** |  |
|  |  | (0.030) | (0.031) |  |
| Extraversion |  | -0.113 | 0.043 |  |
|  |  | (0.074) | (0.146) |  |
| Agreeableness |  | 0.151 | 0.223 |  |
|  |  | (0.077) | (0.155) |  |
| Conscientiousness |  | 0.125 | 0.516*** |  |
|  |  | (0.075) | (0.143) |  |
| Openness |  | 0.054 | 0.188 |  |
|  |  | (0.069) | (0.179) |  |
| Neuroticism |  | 0.060 | 0.247 |  |
|  |  | (0.086) | (0.149) |  |
| Financial#Extraversion |  |  | -0.490* |  |
|  |  |  | (0.198) |  |
| Health#Extraversion |  |  | 0.072 |  |
|  |  |  | (0.185) |  |
| Environment#Extraversion |  |  | -0.295 |  |
|  |  |  | (0.237) |  |
| Financial#Agreeableness |  |  | 0.074 |  |
|  |  |  | (0.216) |  |
| Health#Agreeableness |  |  | -0.134 |  |
|  |  |  | (0.200) |  |
| Environmental#Agreeableness | |  | -0.149 |  |
|  |  |  | (0.276) |  |
| Financial#Conscientiousness | |  | -0.566** |  |
|  |  |  | (0.206) |  |
| Health#Conscientiousness |  |  | -0.528** |  |
|  |  |  | (0.184) |  |
| Environment#Conscientiousness | |  | -0.330 |  |
|  |  |  | (0.229) |  |
| Financial#Openness |  |  | -0.037 |  |
|  |  |  | (0.220) |  |
| Health#Openness |  |  | -0.178 |  |
|  |  |  | (0.209) |  |
| Environmental#Openness |  |  | -0.172 |  |
|  |  |  | (0.226) |  |
| Financial#Neuroticism |  |  | -0.082 |  |
|  |  |  | (0.208) |  |
| Health#Neuroticism |  |  | -0.040 |  |
|  |  |  | (0.181) |  |
| Environmental#Neuroticism | |  | -0.662* |  |
|  |  |  | (0.267) |  |
| N | 303 | 303 | 303 |  |
| Prob > χ² | 0.432 | 0.000 | 0.000 |  |
| Pseudo R² | 0.003 | 0.114 | 0.155 |  |
| Notes: *p < 0.05; **p < 0.01; ***p < 0.001; robust standard errors reported in parentheses. (1), (2), and (3) represent respectively Model 1, Model 2 and Model 3. | | | |  |
|  |  |  |  |  |

**Figure A1: Predicted probabilities by framing condition and openness**

**Figure A2: Predicted probabilities by framing condition and conscientiousness**
